# The effect of plyometric complex training on lower-limb explosive power in adolescents: a systematic review and meta-analysis

**DOI:** 10.3389/fphys.2025.1716568

**Published:** 2025-12-03

**Authors:** Jie Zhang, Xiuling Wu, Xing Ye

**Affiliations:** 1 School of Physical Education and Tourism, Polus International College, Chengdu, Sichuan, China; 2 School of Leisure Sports, Chengdu Sport University, Chengdu, Sichuan, China; 3 School of Sport and Human Movement Science, Beijing Sport University, Beijing, China

**Keywords:** plyometric complex training, adolescents, lower-limb explosive power, meta-analysis, stretch-shortening cycle

## Abstract

**Objective:**

In this meta-analysis, we aimed to examine the effects of plyometric complex training (PT) on lower-limb explosive power in adolescents and explore the moderating role of different training variables.

**Methods:**

Eight databases, including CNKI, PubMed, and Web of Science, were systematically searched. Studies were screened according to predefined inclusion, exclusion, and quality assessment criteria. Data were analyzed using STATA 17.0 and Review Manager 5.4.

**Results:**

A total of 11 studies involving 311 participants were included. The meta-analysis showed that PT significantly improved countermovement jump (CMJ) height (MD = 2.20, 95% CI: 1.46–2.95, P < 0.00001) and squat jump (SJ) height (MD = 2.13, 95% CI: 1.32–2.94, P < 0.00001). For sprint performance, PT yielded significant improvements in 20-m sprint time (MD = −0.10, 95% CI: 0.18 to −0.01, P < 0.05), whereas the improvement in 10-m sprint performance was not statistically significant. Subgroup analysis indicated that an intervention duration of ≥8 weeks was a key factor for achieving significant effects.

**Conclusion:**

PT is useful in improving lower-limb explosive power in teenagers. For the best results, training programs should consider adolescents’ physiological characteristics and use protocols that run for at least 8 weeks, with 2–3 sessions each week, each lasting 20 min–30 min and separated by 1 min–2 min of rest between the sets. The emphasis should be on increasing the jumping ability and short-distance sprint performance.

**Systematic Review registration:**

https://www.crd.york.ac.uk/PROSPERO/myprospero, identifier CRD420251149485.

## 1 Introduction

With the increasing intensity of competitive sports and growing public awareness of health, strength and power training have emerged as key components of adolescent physical development, gaining prominence in athlete development, school-based physical education, and public fitness programs (Channell and Barfield, 2008). Among these capacities, lower-limb explosive power is a critical determinant of performance in activities such as jumping, acceleration, and change-of-direction movements. Scientifically guided training of this capacity is of substantial theoretical and practical value for enhancing adolescents’ athletic performance and preventing sports-related injuries. Plyometric complex training (PT) has gained considerable attention in the field of youth sports due to its potential to improve explosive performance. Multiple randomized controlled trials have demonstrated that PT can significantly enhance key performance indicators in adolescents, such as vertical jump (VJ) height, short-distance sprint performance, and repeated sprint ability (RSA) (Negra et al., 2020; Ramirez-Campillo et al., 2020b; Hernandez-Martinez et al., 2023). For example, 7–9 weeks of PT interventions, conducted twice weekly, effectively improved the VJ height and sprint speed in adolescent soccer players (Ramirez-Campillo et al., 2014; Sáez de Villarreal et al., 2015). Similarly, a 6-week PT program conducted twice per week significantly improved countermovement jump (CMJ), squat jump (SJ), and 20-m sprint performance in adolescent basketball and gymnastics athletes (Aztarain-Cardiel et al., 2024; Hall et al., 2016). A single-blind randomized controlled trial further demonstrated that a 6-week, twice-weekly PT program not only improved CMJ performance in U14 track athletes but also enhanced RSI and leg spring stiffness (LSS), highlighting PT’s dual value in performance enhancement and injury prevention (Cardiel-Sanchez et al., 2024). However, the effects of PT remain inconsistent. Twelve weeks of twice-weekly PT were compared with resistance training and 7 weeks of horizontal and VJ training, showing that although the PT program improved the acceleration ability and muscle strength, no significant changes were observed in the sprint speed or CMJ performance (Sammoud et al., 2022; Ribeiro et al., 2020). Similarly, no significant effects were reported following 8 weeks of low-volume PT in adolescents (Liu et al., 2024). Systematic reviews and meta-analyses, as core evidence-based research methods, have confirmed the benefits of PT in improving the muscle strength, endurance, sprinting, and change-of-direction performance (Chen et al., 2023; Deng et al., 2023; Ramirez-Campillo et al., 2023), often outperforming traditional training methods. Nonetheless, most existing studies focus on the overall effects of PT on athletic performance, whereas targeted analyses in adolescent populations remain limited. In particular, the influence of key training variables, such as intensity, frequency, and duration, has not been comprehensively explored, hindering the development of optimized training programs in practice.

Therefore, in the present study, we conducted a meta-analysis of randomized controlled trials to systematically evaluate the effects of PT on lower-limb explosive power in adolescents. CMJ, SJ, and short-distance sprint performance were selected as the primary outcome measures. In this study, we aim to provide a scientific assessment of PT’s impact on jumping and sprinting performance in adolescents and explore optimal training variables, thereby offering empirical evidence and practical guidance for designing lower-limb explosive power training programs.

## 2 Methods

This study was conducted according to the Preferred Reporting Items for Systematic Reviews and Meta-Analyses (PRISMA) statement and the Cochrane Handbook of Systematic Reviews. Additionally, this review has been registered in the International Prospective Register of Systematic Reviews (PROSPERO) database under the identifier CRD420251149485.

### 2.1 Literature search strategy

The characteristics of the data sources are summarized (Table 1).

**TABLE 1:** Summary of the data sources.

Item	Description
Databases	CNKI, Wanfang, VIP, Web of science, PubMed, Cochrane library, Embase, and SPORTDiscus
Time span	From database inception to May 2025
Search strategy	A combination of subject terms and free-text terms was used for the literature search
Search terms	The search keywords included “plyometrics,” “plyometric training,” “stretch-shortening cycle,” “adolescent,” “lower body,” “power,” and “jump performance”
Document type	Journal article
Number of retrieved records	A total of 581 articles were initially identified

### 2.2 Inclusion and exclusion criteria

The inclusion and exclusion criteria for the studies are presented (Table 2).

**TABLE 2:** Inclusion and exclusion criteria for the studies.

Criteria	Details
Inclusion criteria	1. Study design must be randomized controlled trials (RCTs)
	2. Participants must be healthy adolescents aged 10–19 years, in accordance with the WHO (2006) guidelines for adolescent health
	3. Intervention period ≥4 weeks, with a total of ≥8 training sessions
	4. Experimental group must follow a plyometric training protocol
	5. Studies must include a parallel control group or use other training methods as a comparator
	6. Outcome measures limited to jumping (cm) and sprint (s) performance, consistent with the extraction criteria of this study
	7. Studies must provide sufficient baseline data for calculation (e.g., sample size, mean, and standard deviation)
Exclusion criteria	1. Participants are not adolescents
	2. Non-RCT studies
	3. Studies with missing or incomplete data
	4. Studies for which the full text or research summary cannot be obtained, including theses or duplicate publications

### 2.3 Data extraction and quality assessment

All potentially relevant articles identified from eight databases according to the search strategy were imported into EndNote 21. Duplicates were removed using EndNote and manual screening. Two research workers independently screened the articles, with discrepancies resolved through discussion or, if necessary, by a third research worker. Data extraction was performed independently by two research workers and included the following information: first author and publication year, participant characteristics (sample size, sex, age, and training background), details of the intervention and control protocols (duration, frequency, intensity, and session length), and outcome measures. The quality of the included randomized controlled trials (RCTs) was assessed using the Cochrane Risk of Bias tool across seven domains, with each being rated as low, unclear, or high risk. Assessments were conducted independently by two research workers, and the final risk-of-bias summary was generated using RevMan 5.4.

### 2.4 Statistical analysis

Meta-analyses of the outcome measures from the included studies were performed using STATA 17.0 and Review Manager 5.4. For continuous variables, pooled effect sizes were expressed as the mean differences (MDs). Heterogeneity among studies was assessed using the I^2^ statistic, with I^2^ < 50% indicating low heterogeneity, in which case a fixed-effects (FE) model was applied. When I^2^ > 50%, substantial heterogeneity was considered, and the sources of heterogeneity were further explored through subgroup or sensitivity analyses. If heterogeneity remained high, a random-effects (RE) model was used. Publication bias was evaluated using Egger’s test in STATA 17.0, and the funnel plots were generated in Review Manager 5.4.

## 3 Results

### 3.1 Literature search

**FIGURE 1:**
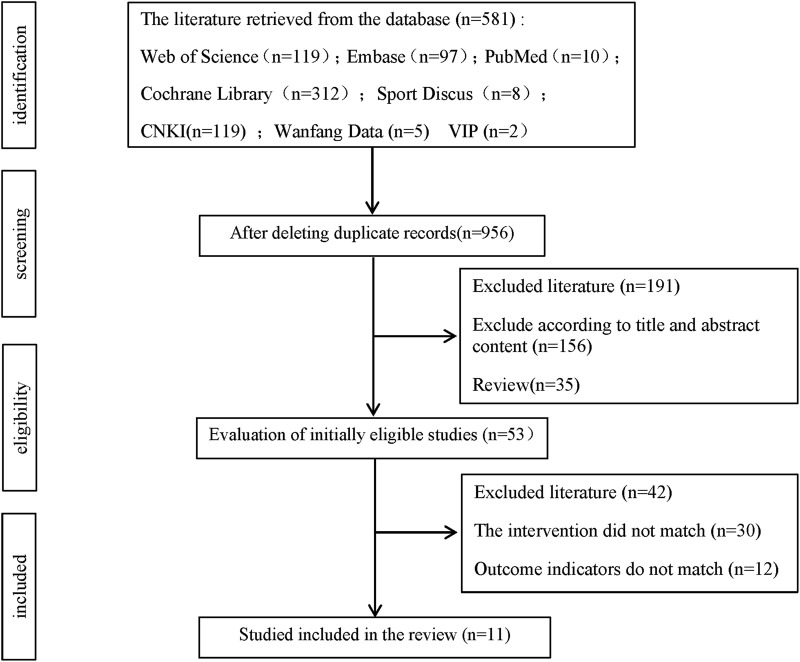
Research screening and selection process according to PRISMA.

A total of 581 records were initially identified through the literature search, including 119 from Web of Science, 10 from PubMed, 312 from Cochrane Library, 97 from Embase, eight from SPORTDiscus, 28 from CNKI, five from Wanfang, and two from VIP. After removing duplicates and irrelevant records, 337 articles were excluded. Based on the screening of titles, abstracts, and study types, a further 191 articles were excluded. Following a full-text review, 42 additional studies were excluded. Ultimately, 11 studies were included in the systematic review and meta-analysis (Figure 1).

### 3.2 Characteristics of studies

A total of 11 studies were included in the present analysis (Tables 3–4), involving 311 participants, of whom 291 were male and 20 were female. The training protocols varied across studies, with intervention durations ranging from 6 to 9 weeks. The duration of individual training sessions was approximately 30 min, with the shortest being 15 min. The training frequency was predominantly twice per week, although some studies reported three sessions per week. The majority of interventions were classified as high-intensity. Lower-limb explosive strength was primarily assessed using CMJ, SJ, and sprint tests over 10 m and 20 m. The study populations mainly consisted of adolescent soccer and basketball players.

**TABLE 3:** Basic characteristics of the included studies.

Researcher	Participants				Experimental group intervention				Outcome
	Sample (n)	Sex	Age (M±SD)	Training background	Intervention duration (weeks)	Training frequency (/week)	Session duration (min)	Intervention intensity	
Chelly et al. (2014)	C = 11 E = 12	Male	17.20 ± 0.4 17.10 ± 0.3	Handball players	8	2	40	Moderate-to-high intensity	SJ and CMJ
Ramírez-Campillo et al. (2014)	C = 38 E = 38	Male	13.20 ± 1.8 13.20 ± 1.8	Soccer players	7	2	20	High intensity	CMJ and sprint
Sáez de Villarreal et al. (2015)	C = 13 E = 13	Male	14.90 ± 0.17 15.33 ± 0.34	Soccer players	9	2	25	Moderate intensity	CMJ and sprint
Cardiel-Sanchez et al. (2024)	C = 10 E = 11	Male	15.30 ± 0.50 15.10 ± 0.20	Basketball players	6	2	20	Low-to-moderate intensity	CMJ, SJ, and sprint
Liu et al. (2024)	C = 17 E = 17	Male	16.30 ± 0.60 16.30 ± 0.60	Soccer players	8	4	15	High intensity	CMJ, SJ, and sprint
Ramírez-Campillo et al. (2020a)	C = 12 E = 12	Male	17.10 ± 0.50 16.90 ± 0.70	Soccer players	7	2	20	High intensity	CMJ and sprint
Hernandez-Martinez et al. (2023)	C = 13 E = 14	Male	15.00 ± 0.85 14.50 ± 0.57	Volleyball players	8	2	25	Low-to-moderate intensity	CMJ, SJ, and sprint
Padrón-Cabo et al. (2021)	C = 10 E = 10	Male	12.39 ± 0.56 12.60 ± 0.70	Soccer players	6	3	35	High intensity	CMJ, SJ, and sprint
Negra et al. (2020)	C = 11 E = 13	Male	12.70 ± 0.20 12.70 ± 0.20	Soccer players	8	2	30	High intensity	Sprint
Hall et al. (2016)	C = 10 E = 10	Female	12.50 ± 1.67 12.50 ± 1.67	Gymnasts	6	2	40	Moderate-to-high intensity	CMJ
Ribeiro et al. (2020)	C = 8 E = 8	Male	18.4 ± 0.52 18.6 ± 0.52	Soccer players	7	2	40	High intensity	CMJ, SJ, and sprint

E: experimental group; C: control group; M: mean; SD: standard deviation; CMJ: countermovement jump; SJ: squat jump; sprint: sprint performance (10 m–20 m).

**TABLE 4:** Plyometric training intervention methods.

Author	Experimental group intervention protocol	Intra-set rest	Inter-set rest
Chelly et al.	0.4 m hurdle/depth jumps: weeks 1–3 (5–10 sets × 10 reps), week 4 (5 × 10), and weeks 5–8 (4 × 10)	30 s	1 min–1.5 min
Ramírez-Campillo et al.	DJ height: 20/40/60 cm (alternated weekly); 2 × 10 reps	15 s	1.5 min
Sáez de Villarreal et al.	Half-squat, vertical, and lateral hurdle jumps: 9 weeks progressive, 2 × 6–8 (W 1–3), 3 × 6–8 (W 4–7), and 4 × 6–10 (W 8–9)	10 s–30 s	1 min
Cardiel-Sanchez et al.	Bilateral vertical jumps: weeks 1–4 progressive (5–8 × 8) and weeks 5–6 tapering (6–5 × 8)	5 s	2 min
Liu et al.	Horizontal, reactive, and triple jumps: bilateral and unilateral variations, 3–5 sets per exercise; includes unilateral 10-cm drop jumps	Right now	3 min
Negra et al.	1 CMJ +1 horizontal jump; 5–8 sets × 10–15 reps; total jumps increased from 50 (week 1) to 120 (week 8)	Right now	3 min
Ramírez-Campillo et al.	Weeks 1–6: progressive; week 7: taper; 13 exercises/session (e.g., wall, squat, tuck, long jump, alt-leg bound, and 20-cm drop)	5 s–15 s	3 min–4 min
Hernandez-Martinez et al.	Depth jump, single-leg CMJ, 180° turn jump, repeated CMJ, and squat jump; weeks 1–2: 16 × 7; week 7: 28 × 7; and week 8: taper (98 total jumps)	30 s	2 min
Padrón-Cabo et al.	Weeks 1–6: progressive jumps: 1–2 two-legged/lateral 5 × 2; 3–4 reverse hopscotch/split 5 × 2→3 × 4; 5–6 modified hopscotch/single-leg lateral 3 × 4→7 × 2	30 s	1 min
Hall et al.	6 weeks, 2×/wk: squat, hurdle, box (repeated/30 cm drop), medicine ball (2 kg–3 kg) chest and single-arm throws	30 s	1 min
Ribeiro et al.	Phase 1–3: backward and forward jumps (1 × 8); single-leg forward jumps, right 2 × 8, left 3 × 12; box jumps onto/off 45 cm and 55 cm boxes (3 × 10)	10 s	2 min

### 3.3 Methodological quality

**FIGURE 2:**
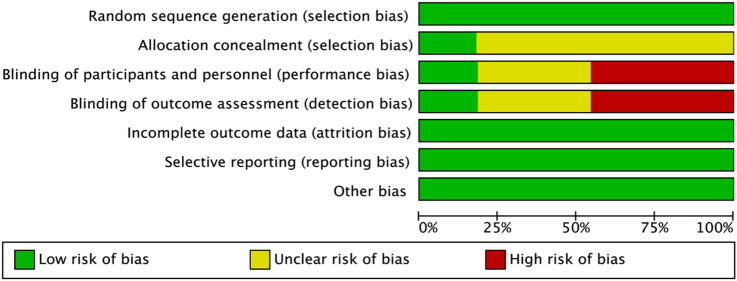
Risk-of-bias graph for the included studies.

**FIGURE 3:**
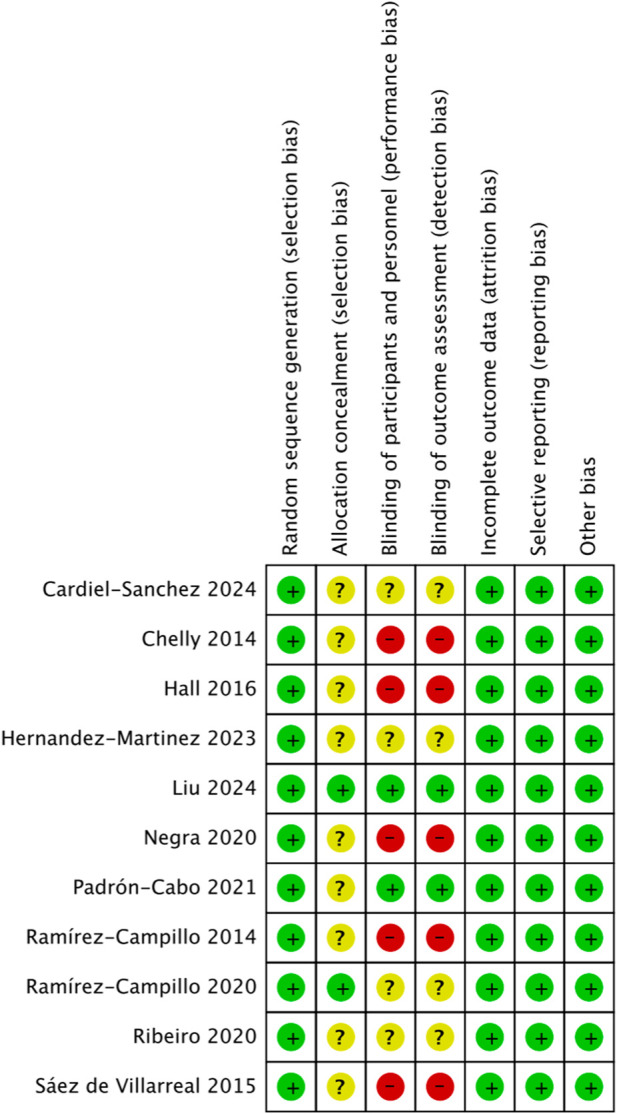
Summary of risk of bias for the included studies.

All 11 studies included in this review used random allocation methods. Regarding allocation concealment, two studies explicitly reported specific concealment procedures, whereas the remaining nine did not provide sufficient details and were, therefore, judged as “unclear.” Concerning blinding, two studies clearly reported the use of blinding, five studies did not use blinding, and four studies provided insufficient details, and thus were classified as “unclear.” None of the studies exhibited missing outcome data, selective outcome reporting, or other evident sources of bias. Overall, the integrity of the outcome data and reporting quality were deemed satisfactory (Figures 2, 3).

### 3.4 Meta-analysis results

#### 3.4.1 Effects of plyometric complex training on countermovement jump performance

**FIGURE 4:**
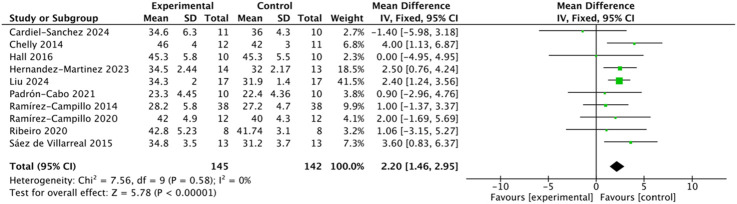
Forest plot of plyometric training effects on countermovement jump (CMJ) performance in adolescents.

A total of 10 studies involving 287 participants reported CMJ as an outcome measure. The heterogeneity test indicated low between-study variability (I^2^ = 0%, Q-test p = 0.58 > 0.10), suggesting no significant heterogeneity; therefore, a fixed-effects model was applied (Figure 4). The pooled analysis demonstrated that the intervention group achieved significantly greater CMJ performance than the control group (MD = 2.20, 95% CI: 1.46–2.95, Z = 5.78, p < 0.000001). Egger’s regression test (p = 0.182 > 0.05) and the symmetrical funnel plot indicated no evidence of significant publication bias.

#### 3.4.2 Effects of plyometric complex training on squat jump performance

**FIGURE 5:**
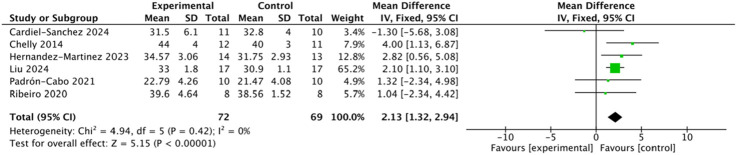
Forest plot of plyometric training effects on squat jump (SJ) performance in adolescents.

Six studies involving 141 participants reported SJ outcomes. Heterogeneity analysis indicated low between-study variability (I^2^ = 0%, Q-test p = 0.42 > 0.10); therefore, a fixed-effects model was applied (Figure 5). The pooled analysis demonstrated that the intervention group exhibited significantly greater SJ performance than the control group (MD = 2.13, 95% CI: 1.32–2.94, Z = 5.15, p < 0.00001). Egger’s regression test (p = 0.599 > 0.05) and the symmetrical funnel plot indicated no evidence of significant publication bias.

#### 3.4.3 Effects of plyometric complex training on 10-m sprint performance in adolescents

**FIGURE 6:**
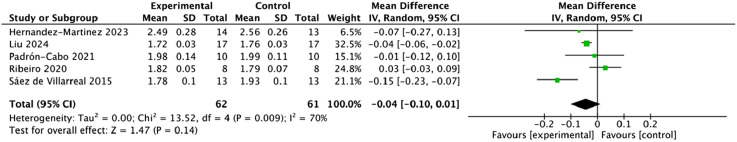
Forest plot of plyometric training effects on 10-m sprint performance in adolescents.

Five studies involving 123 participants reported 10-m sprint outcomes. Heterogeneity analysis indicated substantial between-study variability (I^2^ = 70%, Q-test p = 0.009 < 0.10); therefore, a random-effects model was applied (Figure 6). The pooled analysis showed no significant difference between the intervention and control groups (MD = −0.04, 95% CI: 0.10–0.01, Z = 1.47, p = 0.14), suggesting that PT did not significantly improve 10-m sprint performance in adolescents.

#### 3.4.4 Effects of plyometric complex training on 20-m sprint performance in adolescents

**FIGURE 7:**
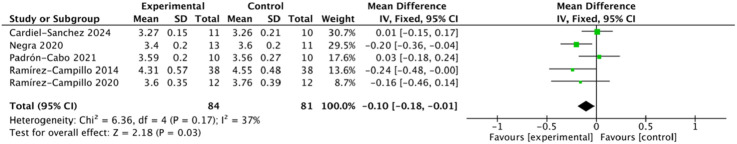
Forest plot of plyometric training effects on 20-m sprint performance in adolescents.

Five studies involving 165 participants reported 20-m sprint outcomes. Heterogeneity analysis indicated low between-study variability (I^2^ = 37%, Q-test p = 0.17 > 0.10); thus, a fixed-effects model was applied (Figure 7). The pooled analysis demonstrated that the intervention group showed significantly better 20-m sprint performance than the control group (MD = −0.10, 95% CI: 0.18 to −0.01, Z = 2.18, p = 0.03). Egger’s regression test (p = 0.678 > 0.05) and the symmetrical funnel plot indicated no evidence of significant publication bias.

### 3.5 Subgroup analysis

#### 3.5.1 Subgroup analysis of plyometric complex training effects on jump performance in adolescents

The overall effect analysis of studies reporting the jump outcomes indicated low heterogeneity (I^2^ = 0%, p = 0.64); therefore, a fixed-effects model was applied. Meta-analysis demonstrated that PT significantly improved jump performance (MD = 2.17, 95% CI: 1.62–2.72, p < 0.00001). Subgroup analyses indicated that the effectiveness of plyometric interventions on jump performance was closely related to the intervention duration. Interventions lasting ≥8 weeks produced significantly greater improvements, with a significant between-group difference (p = 0.01). Different intervention frequencies (1–2 sessions/week vs. 3–4 sessions/week) both significantly enhanced performance, but no significant between-group difference was observed (p = 0.92). Similarly, session durations ≤25 min and >25 min were both effective, with comparable effect sizes. Training intensities ranging from low–moderate to high yielded significant benefits, with slightly higher effect sizes for moderate–high intensity, although the between-group differences were not significant (p = 0.32). Inter-set rest intervals <2 min tended to produce higher effect sizes, but no significant between-group differences were observed (p = 0.63). The findings indicate that the intervention duration is the key factor affecting the outcomes, whereas other variables showed varying trends but did not exhibit significant between-group differences (Table 5).

**TABLE 5:** Subgroup analysis of plyometric training effects on jump performance.

Subgroup	Category	Sample	Effect size	95% confidence interval (CI)	P	I2, %	P-value for heterogeneity
Intervention duration	<8 weeks	6	0.74	−0.48,1.95	>0.05	0	0.97
	≥8 weeks	4	2.17	1.62,2.71	<0.05	0	0.64
Training frequency	1–2 sessions	8	2.20	1.37,3.03	<0.05	6	0.39
	3–4 sessions	2	2.15	1.42,2.87	<0.05	0	0.85
Session duration	≤25 min	6	2.48	1.88,3.08	<0.05	11	0.35
	>25 min	4	2.25	0.92,3.58	<0.05	0	0.52
Intervention intensity	Low–moderate to moderate–high	5	2.58	1.60,3.56	<0.05	26	0.22
	High intensity	5	1.98	1.32,2.64	<0.05	0	0.95
Inter-set Rest interval	<2 min	5	2.42	1.25,3.59	<0.05	4	0.40
	≥2 min	5	2.10	1.48,2.72	<0.05	0	0.64

#### 3.5.2 Subgroup analysis of plyometric complex training effects on sprint performance in adolescents

The overall effect analysis of studies reporting sprint outcomes indicated moderate heterogeneity among studies (I^2^ = 59%, p = 0.009); therefore, a random-effects model was applied. The meta-analysis showed that PT significantly improved the sprint performance (MD = −0.06, 95% CI: 0.11 to −0.01, Z = 2.25, p = 0.02). The negative effect size indicates a significant reduction in sprint time among the participants. Subgroup analyses revealed that interventions lasting ≥8 weeks produced significantly greater improvements in the sprint performance, with a significant between-group difference (p = 0.04), indicating that longer-duration interventions are more effective. Regarding training frequency, both 1–2 sessions/week and 3–4 sessions/week yielded significant benefits, with slightly higher effect sizes for the former, but no significant between-group difference was observed (p = 0.26). Session durations ≤25 min were also effective and tended to outperform longer sessions, although between-group differences were not significant (p = 0.31). Different training intensities and inter-set rest intervals did not show significant effects, and between-group differences were not significant, suggesting that these factors are not the primary determinants of sprint performance. Nevertheless, low–moderate to moderate–high intensity and inter-set rest intervals <2 min exhibited slightly higher effect sizes (Table 6). Based on the integrated findings of jump and sprint performance, it is recommended that PT for adolescents should prioritize intervention duration as the core factor, with the session length as a secondary consideration. Specifically, interventions should last at least 8 weeks, be conducted 2–3 times per week, with each session not exceeding 25 min, at low–moderate to moderate–high intensity, and with inter-set rest intervals shorter than 2 min. This training regimen is expected to effectively enhance the jump or sprint performance in adolescent populations.

**TABLE 6:** Subgroup analysis of plyometric training effects on sprint performance in adolescents.

Subgroup	Category	Sample	Effect size	95% confidence interval (CI)	P	I2	P-value for heterogeneity
Intervention duration	<8 weeks	5	0.16	−0.10, 0.42	>0.05	95%	0.00
	≥8 weeks	4	−0.10	−0.19, -0.02	<0.05	72%	0.01
Training frequency	1–2 sessions	7	−0.09	−0.19, 000	<0.05	70%	0.002
	3–4 sessions	2	−0.04	−0.06, -0.02	<0.05	0%	0.71
Session duration	≤25 min	6	−0.09	−0.16, -0.02	<0.05	55%	0.05
	>25 min	3	−0.03	−0.12, 0.07	>0.05	57%	0.07
Intervention intensity	Low–moderate to moderate–high	3	−0.09	−0.19, 0.01	>0.05	41%	0.18
	High intensity	6	−0.04	−0.10, 0.01	>0.05	54%	0.04
Inter-set Rest interval	<2 min	3	−0.09	−0.19, 0.02	>0.05	57%	0.07
	≥2 min	6	−0.04	−0.09, 0.02	>0.05	50%	0.08

### 3.6 Sensitivity analysis

**FIGURE 8:**
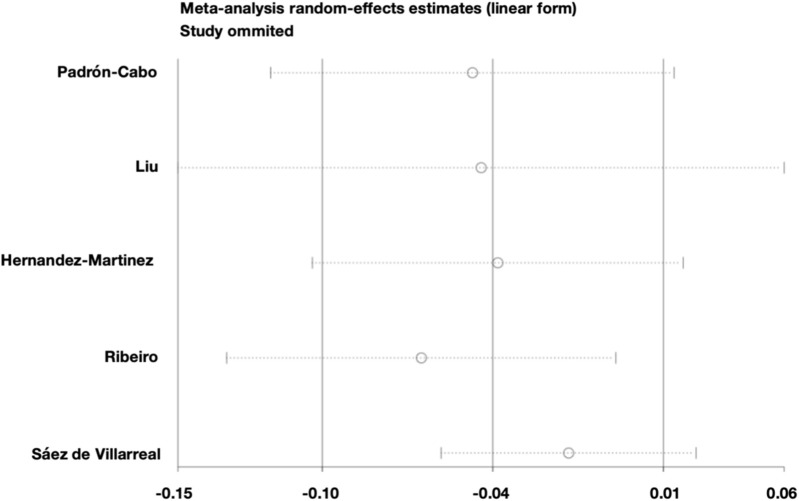
Sensitivity analysis of 10-m sprint performance outcomes.

**FIGURE 9:**
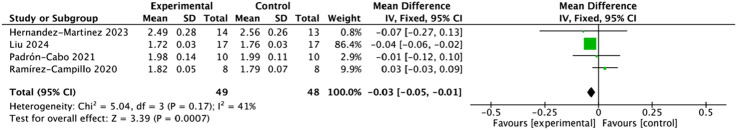
Forest plot of plyometric training effects on 10-m sprint performance in adolescents (fixed-effects model).

The meta-analysis of the 10-m sprint outcomes exhibited substantial heterogeneity (I^2^ = 70%). Sensitivity analysis was performed using a leave-one-out method (Figure 8), indicating that the heterogeneity was mainly attributable to the study by Sáez de Villarreal. After excluding this study, heterogeneity was markedly reduced (I^2^ = 41%, Q-test p = 0.17), and a fixed-effects model was subsequently applied (Figure 9). The pooled effect size indicated that the intervention group significantly outperformed the control group in 10-m sprint performance (MD = −0.03, 95% CI: 0.05 to −0.01, Z = 3.39, p = 0.0007). Egger’s test (p = 0.839 > 0.05) suggested good funnel plot symmetry, indicating no significant publication bias.

### 3.7 Publication bias analysis

**FIGURE 10:**
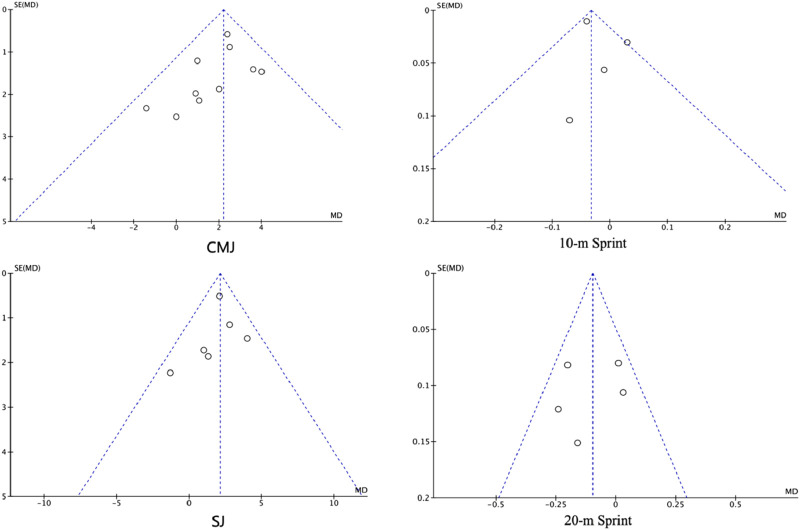
Funnel plots of outcome measures.

As shown in Figure 10, the funnel plots for six studies examining the effects of PT on SJ and five studies examining its effects on 20-m sprint performance demonstrated that the data points were primarily concentrated in the upper–middle region and symmetrically distributed, suggesting no obvious publication bias for these outcomes. For 10-m sprint performance, after excluding the study with high heterogeneity via sensitivity analysis, the remaining four studies also displayed a relatively symmetric distribution, indicating that publication bias was within an acceptable range. Furthermore, Egger’s tests for all four outcomes yielded p > 0.05, supporting the symmetry of the funnel plots and indicating no significant publication bias among the included studies.

## 4 Discussion

In this study, we conducted a meta-analysis to comprehensively evaluate the effects of PT on lower-limb explosive power in adolescents and performed in-depth subgroup analyses of training variables, providing high-level evidence to support the scientific application of PT in the adolescent population.

### 4.1 Jump performance: optimal alignment of stretch-shortening cycle mechanisms with adolescent neural plasticity

The enhancement of jump performance in adolescents induced by PT arises from the specific adaptation of the stretch-shortening cycle (SSC) mechanisms to adolescent neural plasticity, reflecting coordinated adaptations of the neuromuscular-tendinous system (Markovic and Mikulic, 2010; Ramirez-Campillo et al., 2019). Meta-analysis indicated that PT significantly increases CMJ and SJ heights, with effects resulting from multilevel physiological remodeling. At the neural level, adolescents are at the peak of central nervous system plasticity, and PT, through high-velocity eccentric–concentric coupled contractions, accelerates the optimization of motor unit recruitment patterns (Kons et al., 2023; Ramírez-delaCruz et al., 2022). For example, an 8-week PT program was shown to increase CMJ height by 4 cm in adolescent handball players, primarily by shortening the neural conduction delay between muscle stretch perception and concentric force initiation, thereby enhancing the efficiency of elastic energy release (Chelly et al., 2014; Potteiger et al., 1999). At the muscle–tendon level, adolescent tendons have not yet fully matured in stiffness, and the progressive overload design of PT can induce collagen fiber remodeling, significantly increasing tendon stiffness (Kraska et al., 2009). This adaptation reduces energy loss during the concentric phase of the SJ while increasing elastic energy storage during the eccentric phase of the CMJ. Regarding the intervention duration, improvements of jumps are particularly significant when the training period is ≥ 8 weeks. This does not imply that short-term PT is ineffective; rather, the neuromuscular-tendinous system in adolescents requires stepwise accumulation for full adaptation. It has been reported that interventions shorter than 8 weeks primarily activate short-term neural adjustments without inducing long-term tendon remodeling, whereas training lasting ≥8 weeks achieves a cumulative effect of optimized neural control and increased tendon stiffness, transforming transient improvements into stable gains in jump performance (Padrón-Cabo et al., 2021; De Villarreal et al., 2010). These findings also corroborate the conclusion that the effects of exercise interventions accumulate with extended training duration (Gilanyi et al., 2023).

### 4.2 Sprint performance: task-specific differences between 10-m and 20-m sprints

PT significantly improves 20-m sprint performance but shows no significant effect on 10-m sprints. This discrepancy essentially reflects the differing physical capacities required for short sprints of varying distances, highlighting the specific limitations of PT. The 10-m sprint relies primarily on initial acceleration, which is constrained by the athlete’s absolute lower-limb strength and reactive force capacity (Makaruk and Sacewicz, 2010). The efficiency of this phase directly depends on the available maximal strength of the leg extensors, whereas PT primarily enhances power output and SSC efficiency rather than absolute strength. In one study, adolescent soccer players exhibited only a 0.04 s improvement in the 10-m sprint time after 8 weeks of PT, reflecting insufficient development of absolute strength during the starting phase (Liu et al., 2024). Similarly, no difference between the PT and resistance training groups was observed in 10-m sprint performance, further confirming that PT cannot replace strength training in augmenting absolute force (Sammoud et al., 2022). In contrast, the 20-m sprint involves a sustained acceleration phase, during which the performance depends less on absolute strength and more on the ability to maintain power output. A 0.15-s improvement in the 20-m sprint time following 9 weeks of PT in adolescent soccer players has been reported, which can be attributed to the alignment of the sprinting movement pattern with the rapid force generation–energy recycling cycle enhanced by PT (De Villarreal et al., 2015). Moreover, sensitivity analysis showed that after excluding the high-heterogeneity study by Villarreal, the 10-m sprint time also demonstrated a significant improvement, suggesting that discrepancies in 10-m sprint outcomes may be partially due to study-specific training design variations. Overall, the effect of PT on 10-m sprint time remains weaker than that on 20-m sprint time, primarily due to the mismatch between the capacity demands of short-distance sprints and the physiological adaptations elicited by PT.

### 4.3 Optimization of training variables and practical implications

Subgroup analyses clarified the effects of the intervention duration, session time, frequency, and rest intervals on the efficacy of PT, with an intervention period of ≥8 weeks identified as the core threshold, whereas optimization of other variables should consider the physiological tolerance of adolescents. An intervention period of ≥8 weeks is essential for cumulative adaptation. Adolescents possess only 70%–80% of the glycogen reserves of adults and have limited anaerobic capacity, rendering single PT sessions insufficient to induce structural changes in muscles and tendons (Freitas-Junior et al., 2020). Progressive overload over ≥8 weeks allows stepwise adaptations of the neuromuscular-tendinous system while avoiding excessive fatigue (Ramirez-Campillo et al., 2021). Session duration of ≤25 min and inter-set rest of <2 min are critical for maintaining training quality. In one study, the action compliance rate was 92% for 20-min PT sessions but only 71% for 35-min sessions, indicating that shorter sessions enable high-quality training during the “peak attention window,” ensuring effective activation of the SSC mechanism for each repetition and prioritizing quality over quantity (Aztarain-Cardiel et al., 2024; Markovic and Mikulic, 2010). Adolescents exhibit rapid neural excitability decay, and excessively long inter-set rest can lead to loss of muscle pre-activation (Chatzinikolaou et al., 2010). Rest intervals of <2 min help maintain muscle excitability, allowing immediate entry into SSC cycles and improving training efficiency per unit time. It was reported that 1 min–1.5 min rest intervals led to an 18% greater CMJ improvement in adolescent handball players compared to that with 3-min intervals, which was primarily by reducing neural activation delays and maintaining high force quality throughout each set (Chelly et al., 2014). Subgroup analysis also indicated no significant difference between 1–2 sessions and 3–4 sessions per week; however, 2–3 sessions per week appear more suitable for adolescents. In one study, the 4 sessions/week group was effective but not superior to the 2 sessions/week group and carried a higher risk of fatigue accumulation (Pawlik and Mroczek, 2022). Conversely, 2–3 sessions per week allow sufficient muscle recovery, thus preventing declines in movement quality due to inadequate rest (Thomas et al., 2009).

### 4.4 Study positioning and comparison with previous research

The findings of this study are largely consistent with previous reviews (Asadi et al., 2017; De Villarreal et al., 2010; Ramirez-Campillo et al., 2020), indicating that PT is an effective approach for enhancing lower-limb explosive power in adolescents. The underlying rationale for this consensus lies in the high neural plasticity of adolescents, which renders them highly responsive to SSC-based training; compared with adults, adolescents can achieve neuromuscular adaptations over shorter intervention periods. Existing PT-related meta-analyses have predominantly focused on the overall effects, lacking detailed analyses of training variables. The novelty of the present study lies in identifying the critical 8-week intervention threshold through subgroup analyses and further delineating the effects of different training variables, thereby providing direct evidence for the design of precise PT protocols. However, some studies have reported no significant effects of PT on adolescent sprinting or jumping performance, which differs from the present conclusions. This discrepancy can primarily be attributed to task specificity and training design. For example, in one study, no improvement in sprint performance was observed in the PT group compared with resistance training, as the 40-m sprint test used in that study relied more on muscular endurance, whereas PT primarily enhances explosive power rather than endurance (Sammoud et al., 2022). In a 6-week PT protocol, the predominance of low-to-moderate intensity exercises provided insufficient SSC stimulation, and the short intervention duration (<8 weeks) limited structural and neuromuscular adaptations, resulting in negligible performance gains (Padrón-Cabo et al., 2021). These findings corroborate the present conclusion that an intervention period of ≥8 weeks and moderate-to-high intensity are optimal. Collectively, these discrepancies highlight that the effectiveness of PT is not absolute but highly dependent on the alignment between the training design and task demands, thus emphasizing the need for future studies to further refine the correspondence between exercise types and performance outcomes.

### 4.5 Strengths and limitations of the study

In this study, we comprehensively evaluated the effects of PT on multiple lower-limb explosive power outcomes (jumping and sprinting) in adolescents and conducted detailed subgroup analyses to elucidate the moderating roles of training variables, including intervention duration, frequency, session time, intensity, and inter-set rest, providing evidence for the development of precise PT prescriptions. Moreover, adherence to the Cochrane systematic review standards, including rigorous risk-of-bias assessment, sensitivity analysis, and publication bias evaluation, ensured the reliability of the findings. However, several limitations should be acknowledged. The participants were predominantly male football and basketball athletes, with only 20 female subjects, limiting the generalizability of the conclusions to female adolescents and athletes from other sports. Some included studies did not clearly report allocation concealment or blinding procedures, potentially introducing performance or detection bias; although subgroup analyses attempted to account for this, variations in exercise selection and load progression across studies remain. Finally, in this study, we primarily focused on immediate or short-term training effects. Whether the gains in explosive power induced by PT are sustainable over the long term, along with the long-term safety of PT in adolescents, requires further investigation.

## 5 Conclusion

PT can significantly enhance lower-limb explosive power in adolescents, as evidenced by marked improvements in CMJ, SJ, and 20-m sprint performance, with the underlying mechanism attributed to coordinated adaptations of the neuromuscular-tendon system. No significant effects were observed for 10-m sprint performance. An intervention duration of ≥8 weeks was identified as a critical threshold for achieving meaningful outcomes. From a practical perspective, a PT program for adolescents is recommended for the duration of ≥8 weeks, performed 2–3 times per week, for 20 min–30 min per session, with inter-set rest of 1 min–2 min, and at moderate to moderately high intensity, prioritizing the enhancement of jumping ability and 20-m sprint performance. Future research should further explore the differential effects of PT in female adolescents and across various developmental stages (pre-, mid-, and post-puberty), as well as investigate the combined effects and optimal integration strategies of PT with other training modalities, such as resistance or speed training.

## Data Availability

The original contributions presented in the study are included in the article/Supplementary Material; further inquiries can be directed to the corresponding author.

## References

[B1] Asadi A. Arazi H. Ramirez-Campillo R. Moran J. Izquierdo M. (2017). Influence of maturation stage on agility performance gains after plyometric training: a systematic review and meta-analysis. J. Strength and Cond. Res. 31 (9), 2609–2617. 10.1519/JSC.0000000000001994 28557853

[B2] Aztarain-Cardiel K. Garatachea N. Pareja-Blanco F. (2024). Effects of plyometric training volume on physical performance in youth basketball players. J. strength Cond. Res. 38 (7), 1275–1279. 10.1519/JSC.0000000000004779 38900173

[B3] Cardiel-Sanchez S. Rubio-Peiroten A. Molina-Molina A. Garcia-Cebadera Gomez C. Almenar-Arasanz A. Rafales-Perucha A. (2024). Effects of plyometric training on running biomechanics and jumping ability of U14 athletes. J. strength Cond. Res. 38 (11), e656–e663. 10.1519/JSC.0000000000004886 39178063

[B4] Channell B. T. Barfield J. (2008). Effect of olympic and traditional resistance training on vertical jump improvement in high school boys. J. Strength and Cond. Res. 22 (5), 1522–1527. 10.1519/JSC.0b013e318181a3d0 18714236

[B5] Chatzinikolaou A. Fatouros I. G. Gourgoulis V. Avloniti A. Jamurtas A. Z. Nikolaidis M. G. (2010). Time course of changes in performance and inflammatory responses after acute plyometric exercise. J. Strength and Cond. Res. 24 (5), 1389–1398. 10.1519/JSC.0b013e3181d1d318 20386477

[B6] Chelly M. S. Hermassi S. Aouadi R. Shephard R. J. (2014). Effects of 8-week in-season plyometric training on upper and lower limb performance of elite adolescent handball players. J. Strength and Cond. Res. 28 (5), 1401–1410. 10.1519/JSC.0000000000000279 24149768

[B7] Chen L. Huang Z. Xie L. He J. Ji H. Huang W. (2023). Maximizing plyometric training for adolescents: a meta-analysis of ground contact frequency and overall intervention time on jumping ability: a systematic review and meta-analysis. Sci. Rep. 13 (1), 21222. 10.1038/s41598-023-48274-3 38040837 PMC10692103

[B8] De Villarreal E. S.-S. Requena B. Newton R. U. (2010). Does plyometric training improve strength performance? A meta-analysis. J. Sci. Med. sport 13 (5), 513–522. 10.1016/j.jsams.2009.08.005 19897415

[B35] De Villarreal E. S. Suarez-Arrones L. Requena B. Haff G. G. Ferrete C. (2015). Effects of plyometric and sprint training on physical and technical skill performance in adolescent soccer players. J. Strength Cond. Res. 29 (7), 1894–1903. 25635606 10.1519/JSC.0000000000000838

[B10] Deng N. Soh K. G. Abdullah B. Huang D. Xiao W. Liu H. (2023). Effects of plyometric training on technical skill performance among athletes: a systematic review and meta-analysis. PloS one 18 (7), e0288340. 10.1371/journal.pone.0288340 37459333 PMC10351709

[B11] Freitas-Junior C. G. Fortes L. S. Santos T. M. Batista G. R. Gantois P. Paes P. P. (2020). Effects of different training strategies with a weight vest on countermovement vertical jump and change-of-direction ability in male volleyball athletes. J. sports Med. Phys. Fit. 61 (3), 343–349. 10.23736/S0022-4707.20.11171-X 32878424

[B12] Gilanyi Y. L. Wewege M. A. Shah B. Cashin A. G. Williams C. M. Davidson S. R. (2023). Exercise increases pain self-efficacy in adults with nonspecific chronic low back pain: a systematic review and meta-analysis. J. Orthop. and sports Phys. Ther. 53 (6), 335–342–342. 10.2519/jospt.2023.11622 37161890

[B13] Hall E. Bishop D. C. Gee T. I. (2016). Effect of plyometric training on handspring vault performance and functional power in youth female gymnasts. PloS one 11 (2), e0148790. 10.1371/journal.pone.0148790 26859381 PMC4747498

[B14] Hernandez-Martinez J. Guzman-Munoz E. Ramirez-Campillo R. Herrera-Valenzuela T. Magnani Branco B. H. Avila-Valencia S. (2023). Effects of different plyometric training frequencies on physical performance in youth male volleyball players: a randomized trial. Front. physiology 14, 1270512. 10.3389/fphys.2023.1270512 38074324 PMC10701679

[B15] Kons R. L. Orssatto L. B. Ache-Dias J. De Pauw K. Meeusen R. Trajano G. S. (2023). Effects of plyometric training on physical performance: an umbrella review. Sports Medicine-Open 9 (1), 4. 10.1186/s40798-022-00550-8 36625965 PMC9832201

[B16] Kraska J. M. Ramsey M. W. Haff G. G. Fethke N. Sands W. A. Stone M. E. (2009). Relationship between strength characteristics and unweighted and weighted vertical jump height. Int. J. sports physiology Perform. 4 (4), 461–473. 10.1123/ijspp.4.4.461 20029097

[B17] Liu G. Wang X. Xu Q. (2024). Microdosing plyometric training enhances jumping performance, reactive strength index, and acceleration among youth soccer players: a randomized controlled study design. J. sports Sci. and Med. 23 (2), 342–350. 10.52082/jssm.2024.342 38841635 PMC11149064

[B18] Makaruk H. Sacewicz T. (2010). Effects of plyometric training on maximal power output and jumping ability. Hum. Mov. 11 (1), 17–22. 10.2478/v10038-010-0007-1

[B19] Markovic G. Mikulic P. (2010). Neuro-musculoskeletal and performance adaptations to lower-extremity plyometric training. Sports Med. 40 (10), 859–895. 10.2165/11318370-000000000-00000 20836583

[B20] Negra Y. Chaabene H. Fernandez-Fernandez J. Sammoud S. Bouguezzi R. Prieske O. (2020). Short-term plyometric jump training improves repeated-sprint ability in prepuberal Male soccer players. J. strength Cond. Res. 34 (11), 3241–3249. 10.1519/JSC.0000000000002703 33105376

[B21] Padrón-Cabo A. Lorenzo-Martínez M. Pérez-Ferreirós A. Costa P. B. Rey E. (2021). Effects of plyometric training with agility ladder on physical fitness in youth soccer players. Int. J. sports Med. 42 (10), 896–904. 10.1055/a-1308-3316 33592641

[B22] Pawlik D. Mroczek D. (2022). Fatigue and training load factors in volleyball. Int. J. Environ. Res. public health 19 (18), 11149. 10.3390/ijerph191811149 36141425 PMC9517593

[B23] Potteiger J. A. Lockwood R. H. Haub M. D. Dolezal B. A. Almuzaini K. S. Schroeder J. M. (1999). Muscle power and fiber characteristics following 8 weeks of plyometric training. J. Strength and Cond. Res. 13 (3), 275–279. 10.1519/00124278-199908000-00016

[B24] Ramírez-Campillo R. Meylan C. Alvarez C. Henríquez-Olguín C. Martínez C. Cañas-Jamett R. (2014). Effects of In-Season low-volume high-intensity plyometric training on explosive actions and endurance of young soccer players. J. strength Cond. Res. 28 (5), 1335–1342. 10.1519/JSC.0000000000000284 24751658

[B25] Ramirez-Campillo R. Alvarez C. García-Pinillos F. Gentil P. Moran J. Pereira L. A. (2019). Effects of plyometric training on physical performance of young male soccer players: potential effects of different drop jump heights. Pediatr. Exerc. Sci. 31 (3), 306–313. 10.1123/pes.2018-0207 30736711

[B26] Ramirez-Campillo R. Alvarez C. Gentil P. Loturco I. Sanchez-Sanchez J. Izquierdo M. (2020a). Sequencing effects of plyometric training applied before or after regular soccer training on measures of physical fitness in young players. J. strength Cond. Res. 34 (7), 1959–1966. 10.1519/JSC.0000000000002525 29570574

[B27] Ramirez-Campillo R. Sanchez-Sanchez J. Romero-Moraleda B. Yanci J. García-Hermoso A. Manuel Clemente F. (2020b). Effects of plyometric jump training in female soccer player’s vertical jump height: a systematic review with meta-analysis. J. sports Sci. 38 (13), 1475–1487. 10.1080/02640414.2020.1745503 32255389

[B28] Ramirez-Campillo R. García-de-Alcaraz A. Chaabene H. Moran J. Negra Y. Granacher U. (2021). Effects of plyometric jump training on physical fitness in amateur and professional volleyball: a meta-analysis. Front. physiology 12, 636140. 10.3389/fphys.2021.636140 33716784 PMC7952872

[B29] Ramirez-Campillo R. Thapa R. K. Afonso J. Perez-Castilla A. Bishop C. Byrne P. J. (2023). Effects of plyometric jump training on the reactive strength index in healthy individuals across the lifespan: a systematic review with meta-analysis. Sports Med. 53 (5), 1029–1053. 10.1007/s40279-023-01825-0 36906633 PMC10115703

[B30] Ramírez-delaCruz M. Bravo-Sánchez A. Esteban-García P. Jiménez F. Abián-Vicén J. (2022). Effects of plyometric training on lower body muscle architecture, tendon structure, stiffness and physical performance: a systematic review and meta-analysis. Sports Medicine-Open 8 (1), 40. 10.1186/s40798-022-00431-0 35312884 PMC8938535

[B31] Ribeiro J. Teixeira L. Lemos R. Teixeira A. S. Moreira V. Silva P. (2020). Effects of plyometric versus optimum power load training on components of physical fitness in young male soccer players. Int. J. sports physiology Perform. 15 (2), 222–230. 10.1123/ijspp.2019-0039 31094261

[B32] Sáez de Villarreal E. Suarez-Arrones L. Requena B. Haff G. G. Ferrete C. (2015). Effects of plyometric and sprint training on physical and technical skill performance in adolescent soccer players. J. strength Cond. Res. 29 (7), 1894–1903. 10.1519/JSC.0000000000000838 25635606

[B33] Sammoud S. Bouguezzi R. Ramirez-Campillo R. Negra Y. Prieske O. Moran J. (2022). Effects of plyometric jump training versus power training using free weights on measures of physical fitness in youth male soccer players. J. sports Sci. 40 (2), 130–137. 10.1080/02640414.2021.1976570 34749577

[B34] Thomas K. French D. Hayes P. R. (2009). The effect of two plyometric training techniques on muscular power and agility in youth soccer players. J. Strength and Cond. Res. 23 (1), 332–335. 10.1519/JSC.0b013e318183a01a 19002073

